# Inwardly rectifying potassium channels mediate polymyxin-induced nephrotoxicity

**DOI:** 10.1007/s00018-022-04316-z

**Published:** 2022-05-15

**Authors:** Jing Lu, Mohammad A. K. Azad, Julie L. M. Moreau, Yan Zhu, Xukai Jiang, Mary Tonta, Rachel Lam, Hasini Wickremasinghe, Jinxin Zhao, Jiping Wang, Harold A. Coleman, Luke E. Formosa, Tony Velkov, Helena C. Parkington, Alexander N. Combes, Joseph Rosenbluh, Jian Li

**Affiliations:** 1 Infection Program and Department of Microbiology, Biomedicine Discovery Institute, Monash University, Melbourne, VIC 3800 Australia; 2 Development and Stem Cells Program and Department of Anatomy & Developmental Biology, Biomedicine Discovery Institute, Monash University, Melbourne, VIC 3800 Australia; 3 Department of Physiology, Biomedicine Discovery Institute, Monash University, Melbourne, VIC 3800 Australia; 4 Department of Biochemistry and Molecular Biology, Biomedicine Discovery Institute, Monash University, Melbourne, VIC 3800 Australia; 5 Department of Pharmacology and Therapeutics, The University of Melbourne, Melbourne, VIC 3010 Australia; 6 Functional Genomics Platform, Monash University, Melbourne, VIC 3800 Australia; 7 National Glycoengineering Research Center, Shandong University, Qingdao, Shandong 266237 China

**Keywords:** Polymyxin nephrotoxicity, CRISPR/Cas9 screening, Kir4.2, Kir5.1

## Abstract

Polymyxin antibiotics are often used as a last-line defense to treat life-threatening Gram-negative pathogens. However, polymyxin-induced kidney toxicity is a dose-limiting factor of paramount importance and can lead to suboptimal treatment. To elucidate the mechanism and develop effective strategies to overcome polymyxin toxicity, we employed a whole-genome CRISPR screen in human kidney tubular HK-2 cells and identified 86 significant genes that upon knock-out rescued polymyxin-induced toxicity. Specifically, we discovered that knockout of the inwardly rectifying potassium channels Kir4.2 and Kir5.1 (encoded by *KCNJ15* and *KCNJ16*, respectively) rescued polymyxin-induced toxicity in HK-2 cells. Furthermore, we found that polymyxins induced cell depolarization via Kir4.2 and Kir5.1 and a significant cellular uptake of polymyxins was evident. All-atom molecular dynamics simulations revealed that polymyxin B_1_ spontaneously bound to Kir4.2, thereby increasing opening of the channel, resulting in a potassium influx, and changes of the membrane potential. Consistent with these findings, small molecule inhibitors (BaCl_2_ and VU0134992) of Kir potassium channels reduced polymyxin-induced toxicity in cell culture and mouse explant kidney tissue. Our findings provide critical mechanistic information that will help attenuate polymyxin-induced nephrotoxicity in patients and facilitate the design of novel, safer polymyxins.

**Supplementary Information:**

The online version contains supplementary material available at 10.1007/s00018-022-04316-z.

## Introduction

Multidrug-resistant (MDR) Gram-negative bacterial pathogens are a serious global threat to human health [1]. Polymyxins (i.e. polymyxin B and colistin), a group of polycationic lipopeptide antibiotics, are often used as a last-line therapy for MDR Gram-negative bacterial infections [2]. Polymyxins entered the clinic in the late 1950s [2], however, their use declined in the 1970s mainly due to the increasing reports of nephrotoxicity following intravenous administration and the availability of newer, potentially less toxic antibiotics at that time [3]. Over the last two decades, the emergence of resistance to all other available antibiotics and a drying antibiotic discovery pipeline have witnessed a resurgence of using polymyxins to treat life-threatening infections caused by Gram-negative pathogens [4].

Nephrotoxicity is the major dose-limiting factor for intravenous polymyxins and acute kidney injury can occur in up to 60% of patients with the currently recommended dosage regimens [5–7]. Clinical manifestations of polymyxin-induced nephrotoxicity include hypercreatininemia, proteinuria and oliguria [8–10]. Our previous studies found that significant cellular accumulation of polymyxins correlates with activation of death receptor, mitochondrial and endoplasmic reticulum apoptotic pathways, DNA damage and cell cycle arrest [11–13]. Previous pharmacological studies have shown that polymyxins undergo extensive reabsorption by renal tubular cells [14, 15], and several transporters (e.g. PEPT2, megalin, and OCTN2) might contribute to the uptake [9, 12, 16]. However, we currently do not have a detailed molecular understanding of polymyxin-induced nephrotoxicity, and this lack of knowledge has limited the development of strategies for safe and effective administration of polymyxins.

In the present study, we conducted a genome-wide clustered regularly interspaced short palindromic repeats (CRISPR) knockout polymyxin resistance screen and identified 86 significant genes possibly mediating polymyxin toxicity in human kidney cells, amongst which the inwardly rectifying potassium channels were discovered as a major driver. Using cultured cell lines and explant cultured kidneys, we demonstrated that targeting the Kir potassium channels may be an effective approach to overcome polymyxin-induced toxicity in kidney tubular cells.

## Results

### Whole-genome screen identified genes required for polymyxin-induced toxicity in HK-2 cells

**Fig. 1:**
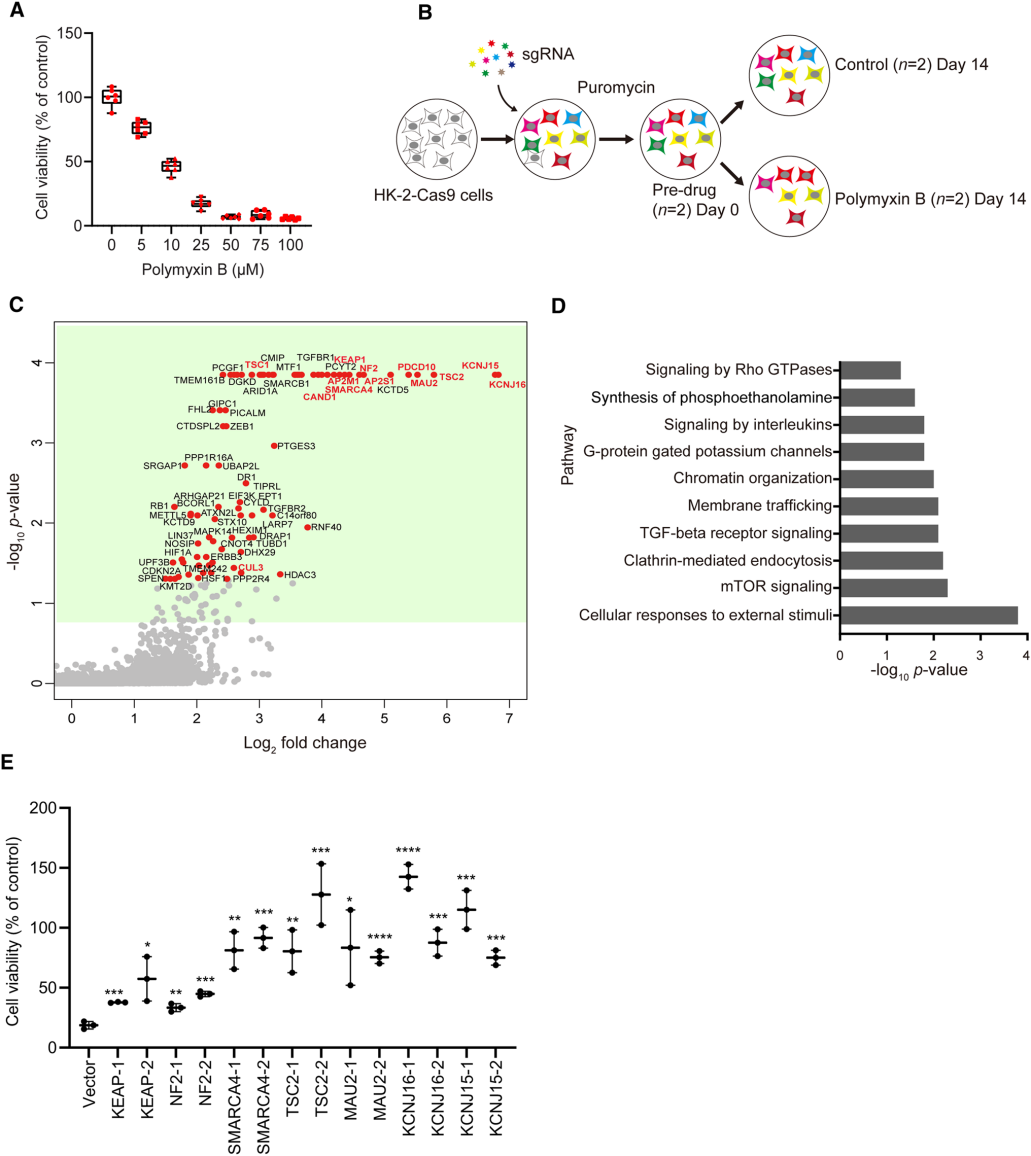
Identification of significant genes mediating polymyxin-induced toxicity in HK-2 cells by CRISPR-Cas9 knockout screen. **A** Viability of HK-2 cells following 24-h treatment with 0–100 µM polymyxin B (*n* = 6). **B** Experimental scheme for CRISPR-Cas9 knockout screen. **C** Volcano plot showing positively selected sgRNAs (red dots in green background, *p* < 0.05) following polymyxin B treatment. The genes discussed in the main text are highlighted in bold and red color. **D** Significantly enriched Reactome pathways (*p* < 0.05). **E** Viability of independent gene knockout cells after polymyxin B treatment. Gene knockout cells were generated by CRISPR editing with guide RNAs. Two sgRNAs were chosen for each gene and are labelled with ‘_1’ and ‘_2’. Viability of gene knockout cells following 25 µM polymyxin B treatment for 24 h were measured with XTT assay (*n* = 3). Two-tailed Student’s *t*-test was used to compare each of the gene knockout groups with the empty vector control. *p < 0.05; **p < 0.01; ***p < 0.001; ****p < 0.0001

Human kidney proximal tubular cell line HK-2 cells have been previously established as a reliable model for nephrotoxicity [16] and are sensitive to polymyxin treatment (Fig. 1A). To define the landscape of genes critical for polymyxin-induced toxicity, we conducted a genome-wide CRISPR knockout polymyxin resistance screen (Fig. 1B). Following transduction of Cas9-expressing HK-2 with a genome-scale single guide RNA (sgRNA) library [17], single gene knock out cells were selected with puromycin for 14 days and the cell mutant library was subsequently treated with 25 µM polymyxin B. After an additional 14 days of polymyxin incubation, DNA was extracted from surviving cells and used for quantification of sgRNA abundance (Fig. 1B). As expected, cells containing sgRNAs targeting known essential genes were depleted from the cell pool (Fig. S1) [18], demonstrating the satisfying reliability of this CRISPR screen. To identify the genes that, upon knockout, induced polymyxin resistance, we used the MAGeCK algorithm [19] to compare sgRNA abundance in HK-2 cells in the absence and presence of polymyxin B treatment. We found 86 genes (false discovery rate [FDR] < 0.05, fold change [FC] ≥ 2) whose knockout conferred resistance to polymyxin B toxicity in HK-2 cells (Fig. 1C and Data file S1). Gene set enrichment analysis (GSEA) showed that these genes were enriched in multiple pathways including potassium channels, responses to external stimuli, mTOR signaling, and endocytosis (*p* < 0.05, Fisher’s exact test; Fig. 1D).

Our screen discovered that perturbations to several complexes induced resistance to polymyxin treatment in HK-2 cells. Specifically, potassium inwardly rectifying channel (Kir) subfamily J member 15 (*KCNJ15*) encoding Kir4.2 and *KCNJ16* encoding Kir5.1, scored as the top hits in this screen. Identification of several key genes in the Akt/mTOR complex, including tuberous sclerosis protein 1 (*TSC1*) and *TSC2* suggested that the Akt/mTOR complex also plays a critical role in polymyxin-induced toxicity. Furthermore, we discovered that loss of endocytic component AP-2 complex subunit sigma (*AP2S1*, FC = 26.0) or AP-2 complex subunit mu (*AP2M1*, FC = 17.1) significantly enhanced polymyxin resistance, indicating that the AP-2 complex mediated endocytosis plays a role in polymyxin toxicity. Consistent with our previous reports showing that polymyxins can cause apoptosis in human kidney proximal tubular and lung epithelial cells [12, 20], we found that knockout of programmed cell death protein 10 (*PDCD10*, inhibiting Akt [21] and modulates apoptosis [22]; 41.2-fold enrichment) had a dramatic effect on polymyxin resistance.

To validate these observations, we generated individual knockout cells for the seven top-hit genes (2 sgRNAs/gene), namely *KCNJ15*, *KCNJ16*, *KEAP1*, *NF2*, *SMARCA4*, *TSC2* and *MAU2*. We confirmed that knockout of these genes contributed to polymyxin B resistance in HK-2 cells (Fig. 1E).

### Transcriptomics in HK-2 cells following polymyxin B treatment supports CRISPR screen results

**Fig. 2:**
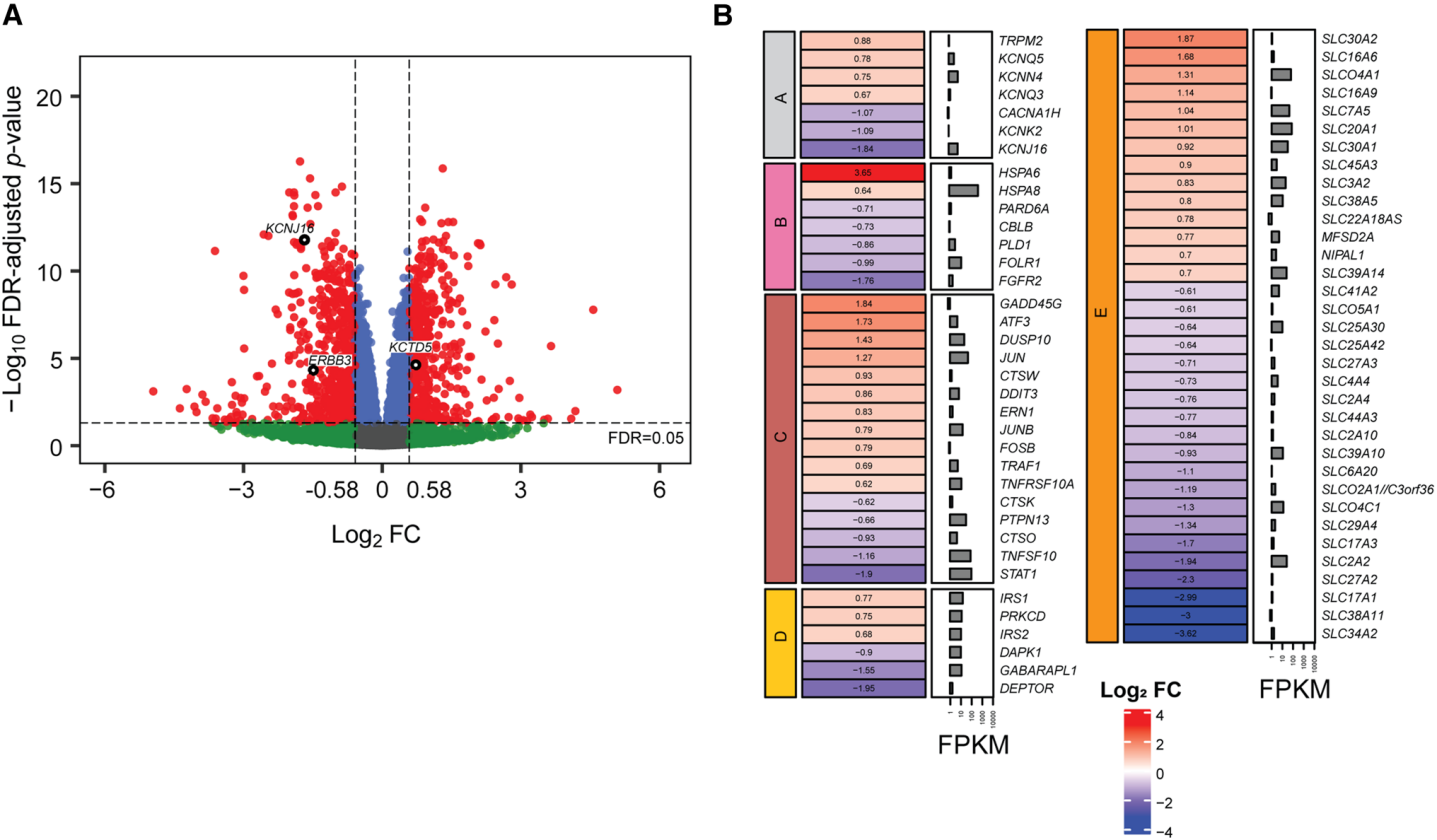
Differential gene expression in HK-2 cells following polymyxin B treatment and pathway enrichment results. **A** Volcano plot showing differentially expressed genes (red) with FDR < 0.05 and FC ≥ 1.5. *KCNJ15*, *KCTD5*, and *ERBB3* were CRISPR identified genes and are labelled in black. **B** Significantly enriched pathways with differentially expressed genes. FPKM: fragments per kilobase of exon per million reads mapped. Pathway names: A, Ion channel; B, Clathrin-dependent endocytosis; C, Apoptosis; D, mTOR; E, SLC transporter family

RNA-seq was conducted to verify the key pathways involved in polymyxin-induced toxicity. Following 24-h treatment with 100 µM polymyxin B, the expression of 1576 genes was significantly changed in HK-2 cells (Fig. 2A and Data file S2). A number of key pathways were significantly affected by the treatment, including ion channels, endocytosis, apoptosis, mTOR and SLC (solute carrier) transporter family (Fig. 2B). Notably, the gene *KCNJ16*, top-ranked in the aforementioned CRISPR screen, was downregulated by 3.6-fold (Fig. 2A). Several genes of clathrin-dependent endocytosis were differentially expressed, including the downregulated *PLD1*, *EPN3* and *DNM3* involved in cargo recruitment and clathrin lattice formation, and upregulated HSC70 chaperone genes that facilitated clathrin coat disassembly. The DEPTOR gene encoding an inhibitor of mTOR was downregulated by 3.9-fold, supporting our CRISPR results that loss of mTOR repressors *TSC1* or *TSC2* improved cell viability following polymyxin B treatment (Fig. 2B). A number of apoptotic genes were significantly changed, including downregulated *MAPK10* (2.5-fold), upregulated *DUSP10* (2.7-fold), upregulated *JUN* (2.4-fold) and *ATF3* (3.3-fold). Significant changes in expression were also observed for genes related to the SLC transporter family (Fig. 2B). Importantly, gene ontology (GO) semantic analysis showed a high-level correlation between the differentially expressed genes and CRISPR-screen identified genes in cellular component (gene cluster semantic similarity = 0.93), molecular function (0.81) and biological process (0.75) (see pairwise gene semantic similarities in Fig. S2).

### *KCNJ15* and *KCNJ16* are essential for polymyxin-induced toxicity in HK-2 cells

**Fig. 3:**
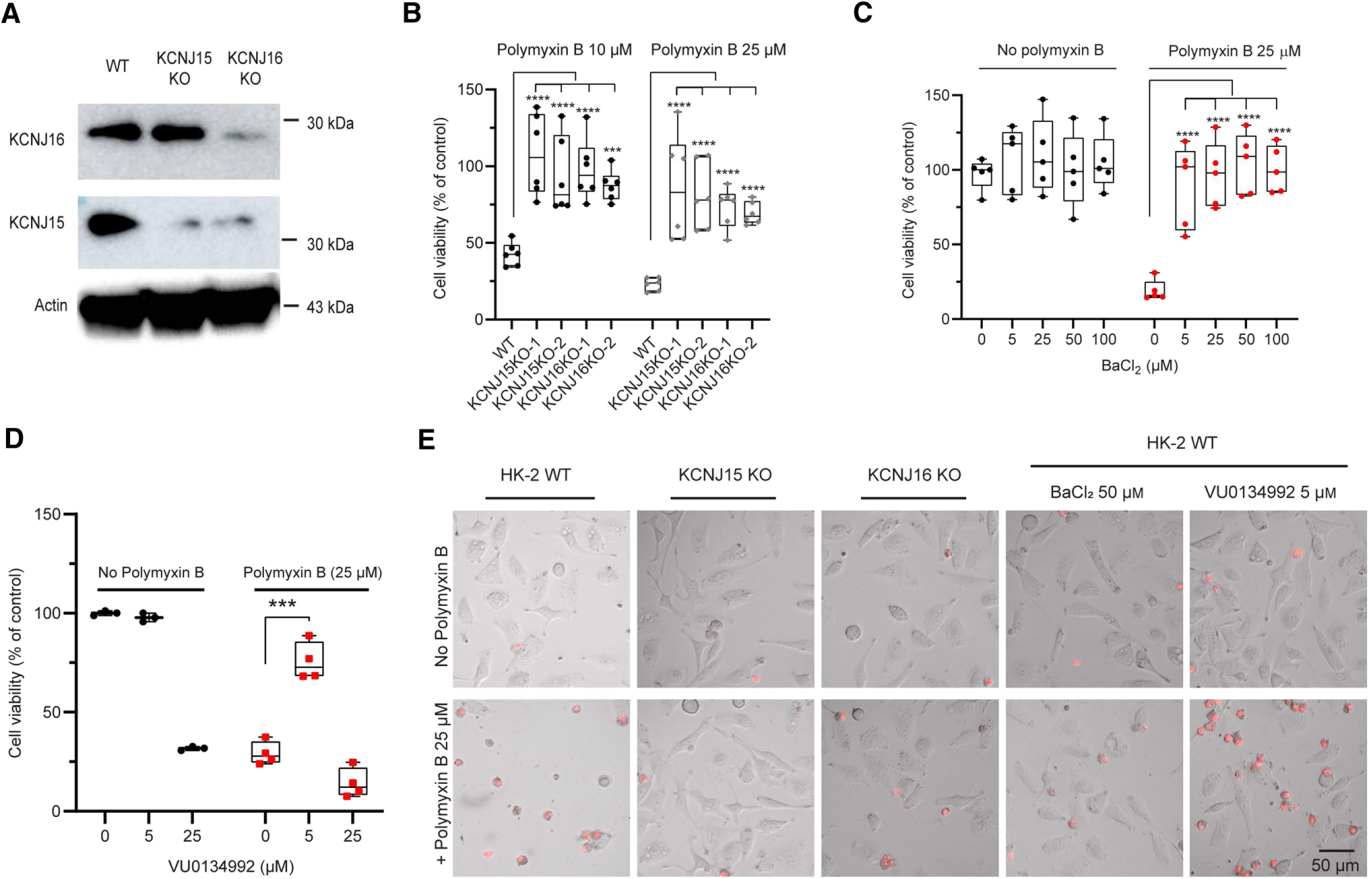
Knockout or inhibition of Kir4.2 and Kir5.1 prevented polymyxin-induced toxicity in HK-2 cells. **A** Western blot showing the expression levels of *KCNJ15* and *KCNJ16* after knockout; actin was used as an internal control. **B** Viability of wild-type HK-2, *KCNJ15* KO and *KCNJ16* KO cells following 24-h exposure to 10 and 25 µM polymyxin B (*n* = 6). **C** Viability of HK-2 cells following 24-h exposure to 0–100 µM BaCl_2_ with or without 25 µM polymyxin B (*n* = 5). **D** Viability of HK-2 cells following the treatment of 0–25 µM VU0134992 alone or in combination with 25 µM polymyxin B for 24 h (*n* = 3 for controls, and *n* = 4 for treatment groups). **E** Morphologies of wild-type, *KNCJ15* KO, and *KCNJ16* KO HK-2 cells with the treatment of 25 µM polymyxin B or polymyxin B with the combination of 50 µM BaCl_2_, or 5 µM VU0134992 to wild-type cells**.** Two-way ANOVA was employed for multi-group comparisons and Tukey's multiple comparison test was employed for post-test. *p < 0.05; **p < 0.01; ***p < 0.001; ****p < 0.0001

The two top scoring genes *KCNJ15* (encoding Kir4.2) and *KCNJ16* (encoding Kir5.1) in our CRISPR screen are both expressed in various kidney cell types [23, 24] and upon knockout a dramatic effect on polymyxin-induced toxicity (FC = 113 and FC = 109, respectively) could occur. Kir channels play a crucial role in maintaining the resting membrane potential [25]. To further explore the roles of both genes, we generated single gene knockout HK-2 cells (Fig. 3A). As Kir5.1 forms heterotetrametric channels with Kir4.2, knockout of Kir4.2 also reduced abundance of Kir5.1 (Fig. 3A). Consistent with our screen result, *KCNJ15* KO and *KCNJ16* KO cells were resistant to the treatment with 10 and 25 μM polymyxin B (Fig. 3B).

Barium chloride (BaCl_2_) is a universal Kir channel inhibitor and blocks the channels at 3–200 µM [26]. Treatment of HK-2 cells with BaCl_2_ (5–100 µM) had no observable effect on cell viability (Fig. 3C). To determine the effect of BaCl_2_ on resistance to polymyxins, we pre-incubated HK-2 cells for 10 min with 5–100 µM BaCl_2_ prior to polymyxin B treatment (25 µM). Polymyxin B induced toxicity was completely rescued by the pre-incubation with 5–100 µM BaCl_2_ (Fig. 3C). The recently discovered Kir4.2 preferable inhibitor VU0134992 [27] at 5 µM also protected cells against 24-h treatment of 25 µM polymyxin B (Fig. 3D). Cell death was evident in wild-type cells following 25 µM polymyxin B treatment for 24 h and the majority of cells were floating and propidium iodide (PI) positive (Fig. 3E). In comparison, cell death was minimal in *KCNJ15* and *KCNJ16* KO cells with or without treatment of 25 µM polymyxin B. Collectively, these results show that Kir4.2 and Kir5.1 play critical roles in polymyxin toxicity in HK-2 cells and the inhibitors (BaCl_2_ and VU0134992) protect cells from polymyxin-induced toxicity.

### Polymyxin B induces cell depolarization in HK-2 cells

**Fig. 4:**
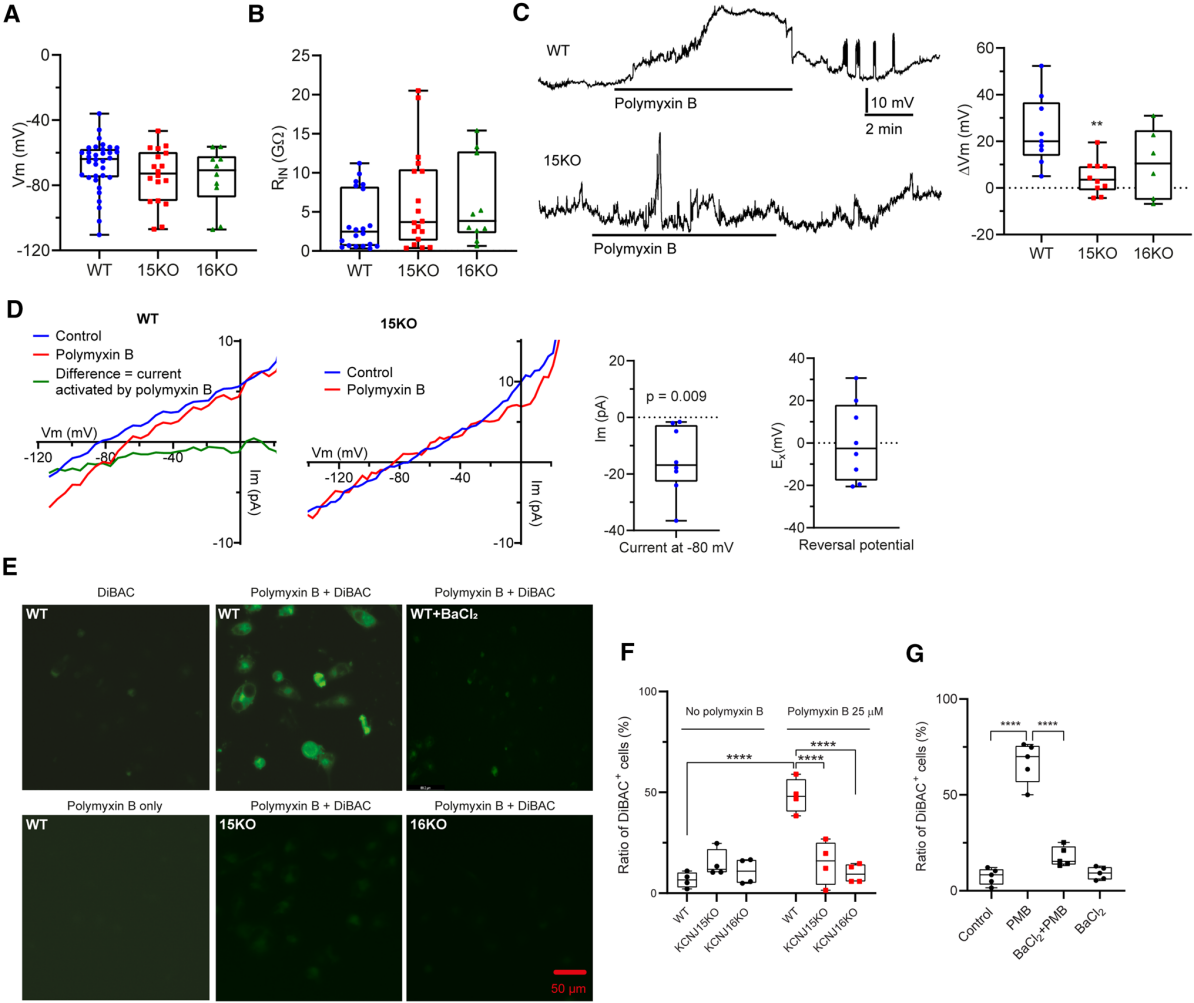
Polymyxin B induced significant electrophysiological changes and membrane depolarization in HK-2 cells. **A** The resting membrane potential in wild-type, *KCNJ15* KO and *KCNJ16* KO cells (*n* = 34, 18 and 10, respectively). **B** Input resistances in wild-type, *KCNJ15* KO and *KCNJ16* KO cells (*n* = 20, 18 and 10, respectively). **C** In current clamp mode, polymyxin B induced approximately 30 mV depolarization in WT cells and this was reversible. Depolarization was not induced in *KCNJ15* KO cells. Polymyxin B induced membrane potential changes are shown aside (*n* = 9, 10 and 7, respectively). **D** In voltage clamp mode, polymyxin B induced a statistically significant inward current (green) in wild-type HK-2 cells (*n* = 8), but not in *KCNJ15* KO cells (*n* = 8). The current and reversal potential values are shown aside. **E** Fluorescent signal detection in HK-2 cells with DiBAC, 25 μM polymyxin B, and DiBAC plus 25 μM polymyxin B. **F** Proportions of DiBAC-positive in wild-type, *KCNJ15* KO, and *KCNJ16* KO HK-2 cells measured by flow cytometry. **G** Proportions of DiBAC-positive HK-2 cells in the control and BaCl_2_ (10 μM) groups with or without 25 μM polymyxin B treatment measured by flow cytometry (*n* = 5 for WT and *n* = 4 for KOs). Data are shown as box and whisker plots. One-way (for WT) or two-way (for KOs) ANOVA was employed for multi-group comparisons. **p < 0.01; ****p < 0.0001

Kir channels are involved in K^+^ homeostasis and play a major role in determining membrane potential in many cell types, including renal epithelial cells [28]. Considering that Kir4.2 and Kir5.1 mediated polymyxin-induced toxicity (Fig. 3) and the differential expression of several voltage-gated channels was evident (Fig. 2B), we hypothesized that polymyxin-induced nephrotoxicity involved disruption of K^+^ homeostasis and cell membrane polarization. Therefore, we employed patch-clamp electrophysiology in wild-type, *KCNJ15* KO and *KCNJ16* KO HK-2 cells. The resting membrane potentials were not significantly different across all three groups (Fig. 4A). The input resistance of HK-2 cells was very high but consistent between groups (Fig. 4B), suggesting that a small current would exert a large effect on membrane potential. In ‘current clamp mode’, polymyxin B (50 µM) induced a significant reversible depolarization (24.5 ± 5.0 mV, *p* = 0.006) in wild-type cells (Fig. 4C). However, this depolarization did not occur in *KCNJ15* KO or *KCNJ16* KO cells (*KCNJ15* KO 4.7 ± 2.3 mV, *p* = 0.238; and *KCNJ16* KO 10.6 ± 6.1 mV, *p* = 0.147; Fig. 4C). Next, the current/voltage (IV) relationship of the membrane was determined by ramping or stepping the membrane from − 120 to + 20 mV in voltage clamp mode. Treatment of HK-2 cells with 50 µM polymyxin B induced an inward current at negative membrane potentials which reversed close to 0 mV (green trace in Fig. 4D). This inward current did not occur in *KCNJ15* KO cells (Fig. 4D) nor in the presence of BaCl_2_ (data not shown). At − 80 mV, the polymyxin B induced current had an amplitude of – 15.2 ± 4.2 pA (significantly different from the control) and a reversal potential of 0.6 ± 6.1 mV (Fig. 4D). These results further confirmed that polymyxin B induced an inward current and cell depolarization in wild-type but not *KCNJ15*/*16* KO cells.

Using a voltage-sensitive dye Bis(1,3-dibutylbarbituric acid) trimethine oxonol (DiBAC) as an independent approach, we observed significant membrane depolarization in HK-2 cells following 1-h treatment with 25 μM polymyxin B, but not in *KCNJ15* KO or *KCNJ6* KO cells (Fig. 4E). Flow cytometry showed that 50.0% of cells were DiBAC-positive following 1-h polymyxin B treatment, whereas polymyxin did not cause significant changes in *KCNJ15* KO or *KCNJ6* KO cells (Fig. 4F). After 24 h, 70.0% of cells were DiBAC-positive following polymyxin B treatment, compared with 7.4% in the untreated group, polymyxin B induced membrane depolarization was alleviated to 17.8% by 10-min pre-treatment with 10 µM BaCl_2_ (Fig. 4G). Taken together, these results demonstrate that in HK-2 cells polymyxin-induced toxicity involves the disruption of K^+^ homeostasis and depolarization.

### Polymyxin B bound and opened Kir4.2 in molecular dynamics simulations

**Fig. 5:**
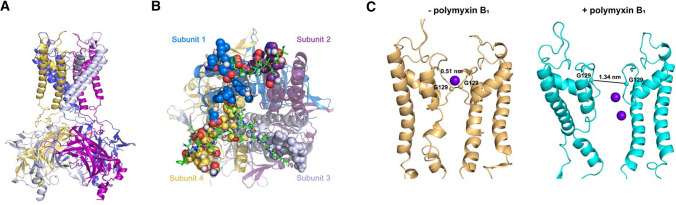
Molecular models of Kir4.2 channel with polymyxin B_1_. **A** The Kir4.2 channel model is shown in NewCartoon presentation (subunits are in yellow, blue, grey and purple). **B** Binding of polymyxin B_1_ to the Kir4.2 channel. Polymyxin B_1_ are shown in green, and the binding amino acids are in red. **C** Gate distance of the channel in the inactivate state (orange) and the state after polymyxin B_1_ bound. Purple balls represent K^+^ atoms. Independent simulations were conducted three times

We used a homology structure model of Kir4.2 channel (Fig. 5A) and polymyxin B_1_ to perform all-atom molecular dynamics simulations of their interactions. Polymyxin B_1_ molecules spontaneously bound to the extracellular region of Kir4.2 that was embedded in a dipalmitoylphosphatidylcholine (DPPC) bilayer (Fig. 5B). Specifically, the key residues responsible for the recognition and binding of polymyxin B_1_ were identified based on their minimum distance (less than 0.4 nm) from polymyxin B_1_, including 96Q, 97L, 98G, 99E, 100S, 101 N, 102S, 103 N, 109 M, 110 K, 111 V, 112D, 113S, 130Y, 131G, 132 V, 134S, 135I, 137E and 138E (Fig. 5B). The key structural moieties of polymyxin B_1_ scaffold in this interaction were also elucidated, i.e. the fatty acyl group, Thr2, Dab3, Dab8 and Dab9 (Fig. 5B). Notably, we discovered that the interaction between polymyxin B_1_ and Kir4.2 increased the open probability of the channel. In the absence of polymyxins, the average distance between the loops of the channel extracellular gate was only approximately 0.51 nm, while it dramatically increased to 1.34 nm in the presence of polymyxin B_1_ (Fig. 5C). Our findings from all-atom molecular dynamics simulations indicate that polymyxins directly interact with the Kir4.2 channel and induce its open-state conformation.

### Inhibition or knockout of *KCNJ15/16* prevented uptake of polymyxin B in HK-2 cells without affecting its antibacterial activity

**Fig. 6:**
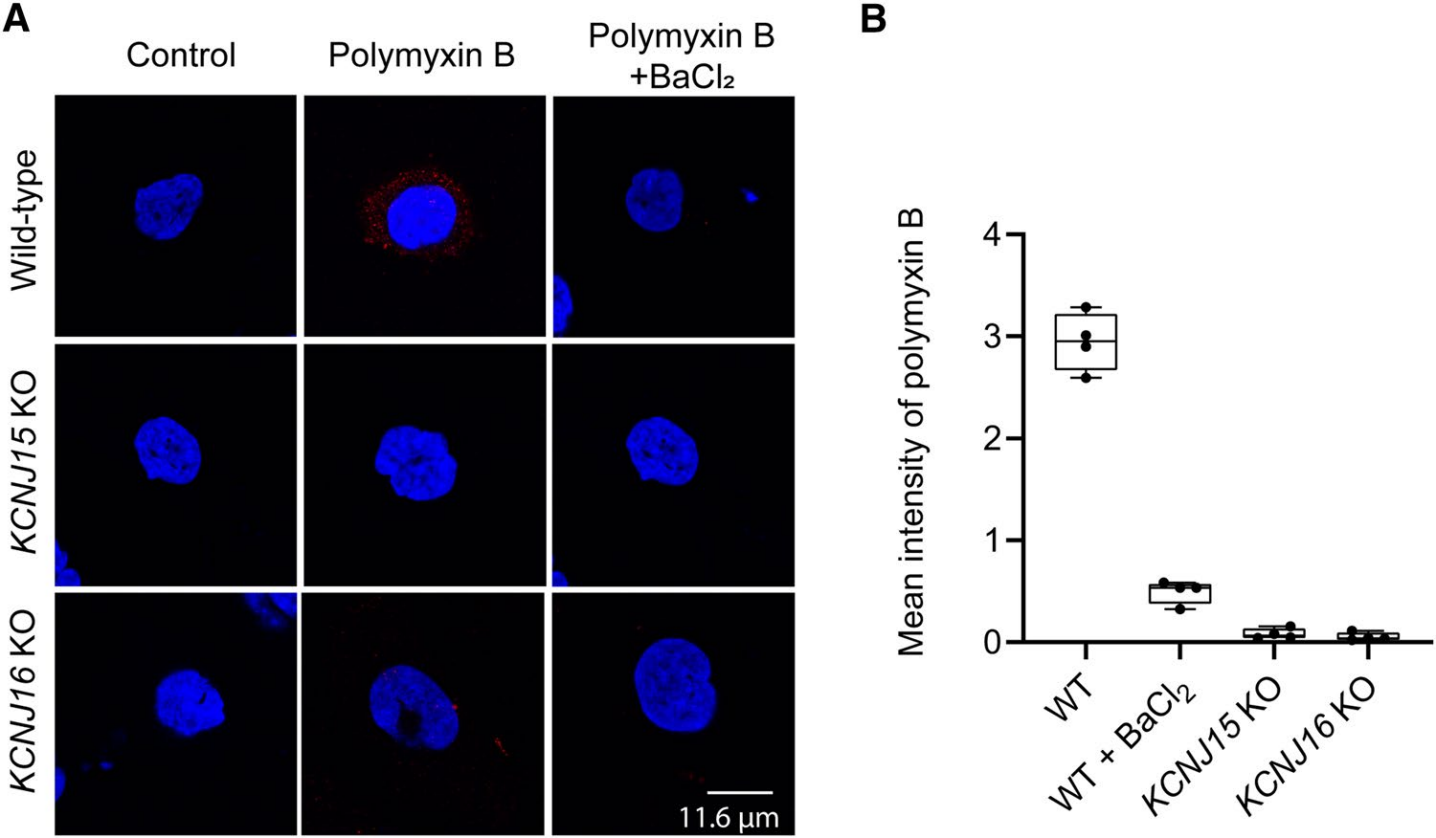
Intracellular accumulation of polymyxin B in wild-type, *KCNJ15* KO and *KCNJ16* KO HK-2 cells with the treatment of 25 µM polymyxin B and 50 µM BaCl_2_ for 6 h.** A** Polymyxin B was immunostained with polymyxin antibody and visualized using Alexa Fluor-594 dye (red). The nucleus was counterstained with DAPI (blue). **B** The plots showing mean fluorescence intensities from each group. The value from control group has been deducted and the mean value from each replicate was plotted (*n* = 4)

As polymyxin intracellular accumulation is indispensable to its toxicity, we evaluated polymyxin B uptake in HK-2 cells using a polymyxin-specific monoclonal antibody [13]. Our result showed that the intracellular accumulation of polymyxin B was blocked by either BaCl_2_ or knockout of *KCNJ15* or *KCNJ16* (Fig. 6). These results demonstrated that *KCNJ15* and *KCNJ16* played critical roles in the cellular uptake of polymyxin B in HK-2 cells and reduction of intracellular uptake prevented its toxicity (Fig. 3).

Subsequently, we assessed whether this pharmaceutical intervention affected the antibacterial activity of polymyxins against three critical Gram-negative pathogens, *Pseudomonas aeruginosa*, *Acinetobacter baumannii* and *Klebsiella pneumoniae*. In the presence of 50 µM BaCl_2_ or 5 µM VU0139942, the antibacterial activity of polymyxin B did not change (Table S1). This result demonstrated that Kir inhibitors can rescue polymyxin-induced kidney toxicity without compromising the antibacterial activity of polymyxins.

### Kir inhibitors rescued polymyxin-induced cell apoptosis and cell death in kidney culture

**Fig. 7:**
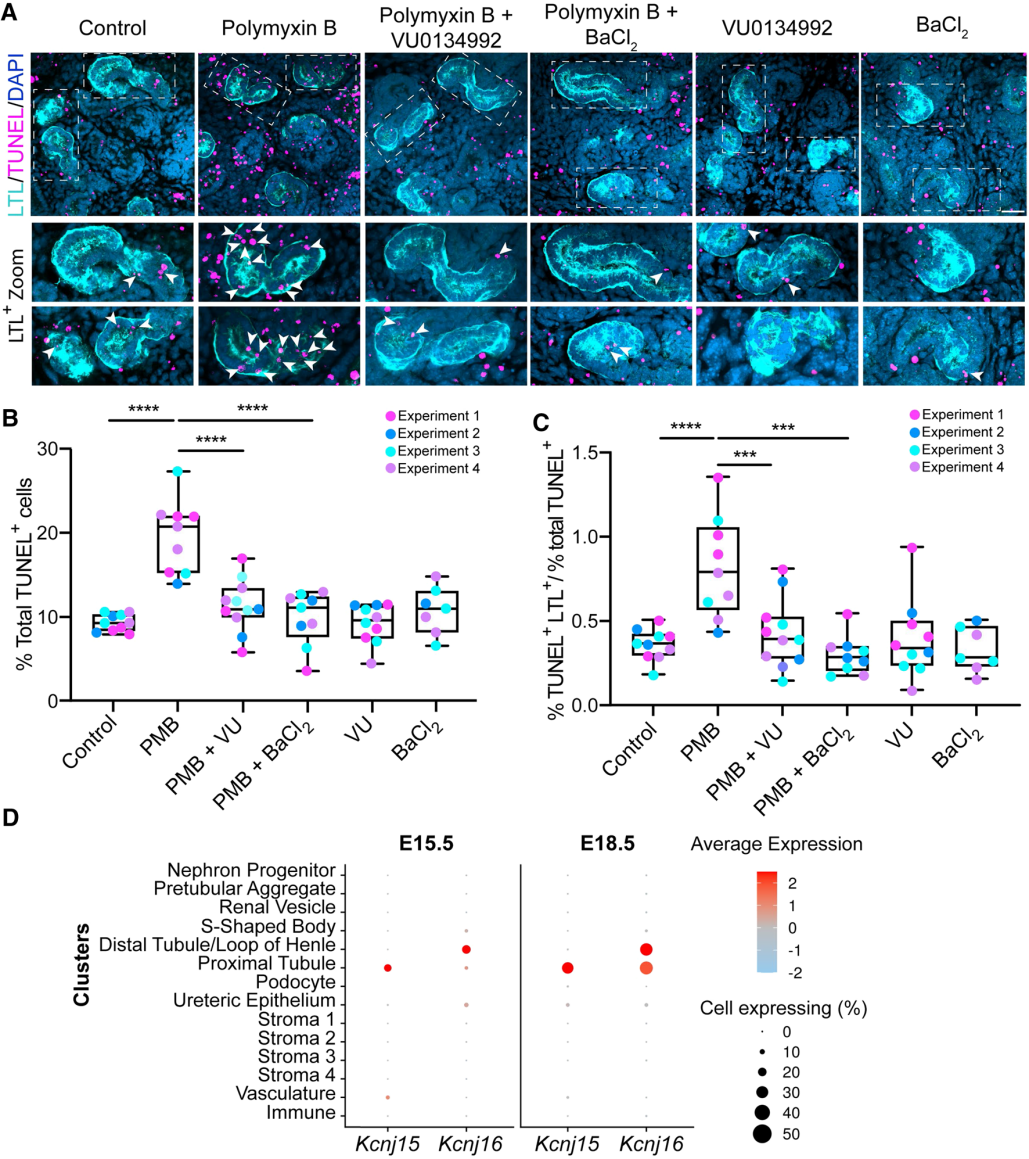
Polymyxin-induced toxicity in mouse kidney explant cultures with or without Kir inhibitors. **A** TUNEL staining (magenta) of explanted kidneys labelled with LTL (proximal tubules, cyan) and DAPI (nuclei, blue) after treatments with 50 μM polymyxin B (PMB), 5 μM VU0134992 (VU) and 50 μM BaCl_2_ alone or in combination. Scale bar = 30 μm. Dashed boxes in the top image panel indicate magnified proximal tubule regions shown below. TUNEL^+^ LTL^+^ cells are marked with arrowheads. **B** Quantification of polymyxin-induced apoptotic cells relative to the total number of cells in each sample (*n* = 4). ****p < 0.0001. **C** Assessment of relative levels of polymyxin-induced apoptosis in tubules with or without polymyxin treatment (*n* = 4). ***p < 0.001. **D** Expression levels of *Kcnj15* and *Kcnj16* in mouse developmental kidneys on embryonic day E15.5 and E18.5. Average gene expression levels (blue-red) and the percentage of cells within a cluster that expressed the gene (circle size) are displayed according to the legends

Our earlier results showed that Kir channels mediated polymyxin nephrotoxicity by enabling an influx of this toxic antibiotic into HK-2 cells. We further examined the ability of Kir channel inhibitors to suppress polymyxin-induced toxicity using mouse kidney explant culture, a 3D tissue model containing living nephrons [29, 30]. Based on the cell culture data (Fig. 3B, C), the protective effect of 50 µM BaCl_2_ and 5 µM VU0134992 was examined in the kidney explant. Propidium iodide staining in explant culture after 24-h polymyxin B treatment (25, 50 and 100 µM) showed that kidney toxicity was induced in a concentration-dependent manner and was suppressed by pre-treatment with the universal Kir inhibitor BaCl_2_ (50 µM) or Kir4 inhibitor VU0134992 (5 µM) (Fig. S3). We used the TUNEL assay and 3D confocal microscopy to quantify polymyxin-induced apoptosis (Fig. 7A–C). Apoptosis occurred in 19.7 ± 4.3% of kidney cells following 24-h polymyxin B treatment, significantly higher than the 9.4 ± 1.0% in the untreated controls (Fig. 7A, B). The Kir inhibitors BaCl_2_ (50 µM) and VU0134992 (5 µM) markedly reduced apoptotic cell death to a level (9.9 ± 3.2% for BaCl_2_ and 11.4 ± 3.0% for VU0134992) similar to that of the untreated controls, demonstrating a protective effect by both inhibitors (Fig. 7B). We previously showed that polymyxins cause apoptosis in proximal tubular cells [7, 13]. Therefore, we tested whether this also occurred in the kidney explant culture and quantified apoptotic cells in proximal tubular (LTL^+^) in the explant cultures. Compared to the untreated control, polymyxin B treatment resulted in two-fold higher cell apoptosis in proximal tubular cells, which was again significantly reduced by supplementation with BaCl_2_ or VU0134992 (Fig. 7C). Single-cell RNA-sequencing data confirmed the co-expression of *Kcnj15* and *Kcnj16* in proximal tubules at the embryo stages of E15.5 (similar to the experimental stage of the kidney explant culture) and E18.5 (Fig. 7D). Overall, these results support a key role of *KCNJ15* and *KCNJ16* in polymyxin-induced apoptosis in kidney tissue and the protective effects of their inhibitors.

## Discussion

Current dosage regimens of the last-line therapy polymyxins are suboptimal [31], primarily because their nephrotoxicity precludes dose escalation and pharmacokinetic/pharmacodynamic optimization against Gram-negative ‘superbugs’ [14, 32]. Previous studies suggest that significant accumulation of polymyxins in kidney proximal tubular cells might lead to the nephrotoxicity [14, 15, 45, 46]; however, the precise mechanism of polymyxin-induced nephrotoxicity remains unclear. In the present study, we employed CRISPR-Cas9 genome editing to generate a genome-wide gene knockout library of HK-2 cells and screened the knockouts for resistance to polymyxin-induced cell death. Unexpectedly, we discovered that the inwardly rectifying potassium channels Kir4.2 (KCNJ15) and Kir5.1 (KCNJ16) mediated polymyxin uptake and toxicity in HK-2 cells.

Individual knockout of *KCNJ15* or *KCNJ16* in HK-2 cells attenuated polymyxin-induced membrane depolarization, reduced intracellular accumulation of polymyxin B, and significantly increased resistance to polymyxin B-induced toxicity (Figs. 3 and 6). Several Kir channel inhibitors/regulators protected HK-2 cells from polymyxin-induced toxicity. In the present study, 10-min incubation with a Kir channel inhibitor BaCl_2_ prior to polymyxin B exposure completely protected cells from polymyxin-induced cell death (Fig. 3C). Kir5.1 forms a heterotetramer with either Kir4.1 or Kir4.2 [28]. Kir4.2 and Kir4.1 shares 60% similarity in the structure [33], while the expression level of Kir4.1 was negligible in HK-2 cells as shown by RNA-seq (Data File S2); nevertheless, we do not exclude similar responses to polymyxin B where Kir4.1 is expressed. Inward/outward flow can be prevented when Ba^2+^ binds to the inside of the Kir4.2 or Kir5.1 pore and blocks the channels [25].

The above hypothesis was verified by our electrophysiological experiments which showed that polymyxin B activated a channel carrying an inward current (Fig. 4D) likely as a result of modulating Kir activity upon binding. Although the current was small, it drastically altered membrane potential within minutes (Fig. 4C, E-G) owing to the high input resistance of these cells. For example, in a 5 GΩ cell such as HK-2 (Fig. 4B), a membrane current of 1 pA would change the membrane potential by 5 mV. This explains the apparent ‘noisiness’ of the membrane potential traces in Fig. 4C, including the transient depolarizing events in both traces. As expected, knocking out *KCNJ15* or *KCNJ16* (Fig. 4C), or addition of Ba^2+^ (Fig. 4F) counteracted this effect and attenuated polymyxin B-induced membrane depolarization. Here, we also employed all-atom molecular simulations [34] to unveil the molecular nature of polymyxin B binding to the potassium channel Kir4.2. As the crystal structure of Kir4.2 has not been published, a homology model of Kir4.2 was built based on the existing crystal structure of a closely related Kir2.2 channel (PDBID:3SPI) [35]. Interestingly, spontaneous binding of polymyxin B dramatically increased opening of the channel (Fig. 5C), which very likely led to the initial instant inward flow of potassium (Fig. 4D).

Nephrotoxicity has been the major safety concern for many drugs in the clinic, including polymyxins. Kidney cells contain a large number of specialized ion channels and transporters acting in concert to reabsorb useful substances from glomerular filtrate and drugs can also be reabsorbed by these transporters [36]. Voltage-gated potassium and calcium channels (*KCNN4*, *KCNK2*, *KCNQ3*, *KCNQ5* and *CACNA1H*) were significantly downregulated in human kidney HK-2 cells following polymyxin B treatment (Fig. 2B), indicating an adaptive cellular response attempting to limit uptake of this toxic peptide. Intracellular concentrations of polymyxins in HK-2 cells can reach ~ 2000 to 5000 times higher than extracellular concentrations [16]. At least three transporters megalin [37], PEPT2 [38] and OCTN2 [39] have been shown to play a key role in polymyxin uptake. However, none of these transporters was identified in our CRISPR screen. The expression of megalin was below the detection limit in the RNA-seq results (Data file S2); while PEPT2 and OCTN2 very likely can compensate for each other if only one transporter lost its function. For the first time, we discovered that polymyxin uptake was affected by Kir functionality given that knockout of *KCNJ15* or *KCNJ16*, or pre-treatment with Kir inhibitor BaCl_2_ led to an almost complete reduction in intracellular accumulation of polymyxin B (Fig. 6). These results also experimentally confirmed that intracellular accumulation of polymyxins leads to apoptosis and cell death [16]. Membrane depolarization likely serves as a primary response to polymyxin treatment and the state of many transporters and ion channels may change as a result of the altered membrane potential (Fig. 4) [36].

Apart from Kir channels, polymyxin-induced toxicity in HK-2 cells possibly involves endocytosis-mediated uptake (Fig. 1D). This is supported by our CRISPR and transcriptomic data (Figs. 1C and 2B). Notably, *AP2S1* and *AP2M1*, encoding the key subunits AP-2, were top-ranked in our CRISPR screen; this is the first time that they have been shown to be associated with polymyxin-induced toxicity. Additionally, a number of other clathrin-dependent endocytic genes were identified by our CRISPR screen and transcriptomics, particularly those involved in initial cargo recruitment, clathrin lattice formation and endosome trafficking (Figs. 1D and 2B). We have previously shown that polymyxin B is co-localized with early endosomes in lung epithelial cells A549 [40]. Taken together, these results suggest that AP-2 mediated clathrin-dependent endocytosis might contribute to polymyxin uptake in human kidney tubular cells. Since knockout of *KCNJ15* or *KCNJ16*, or pre-treatment with BaCl_2_ substantially reduced polymyxin B accumulation in HK-2 cells (Fig. 6), we postulate a crosstalk between Kir channels and endocytosis in polymyxin B uptake that warrants further investigation. In addition, our CRISPR screening showed that knockout of mTOR repressors *TSC1*, *TSC2* and *NF2* enhanced viability of HK-2 cells (Figs. 1C and 2B). mTOR is a major regulator of cellular activities, including microtubule organization, lipolysis, lipid biosynthesis, autophagy and cell proliferation [41]. Our results indicate that, when exposed to polymyxins, activation of mTOR is vital for cell survival [40].

Finally, our proof-of-concept study using mouse kidney explant confirmed in proximal tubules the co-expression of *Kcnj15* and *Kcnj16* at the embryo stages of E15.5 and polymyxin-induced substantial toxicity (Fig. 7). Importantly, Kir inhibitors VU0134992 and BaCl_2_ significantly attenuated polymyxin-induced toxicity in proximal tubules of the mouse kidney explant which is close to the embryo stage of E15.5 (Fig. 7). Both *Kcnj15* (*KCNJ15*) and *Kcnj16* (*KCNJ16*) are highly expressed in proximal tubule in the kidneys of adult mice [42], rats [43] and humans [44]. Previous studies have shown that polymyxins mainly accumulated in kidney proximal tubular cells in rats and mice [45, 46]. Collectively, our cell culture and mouse explant data confirm that significant intracellular accumulation of polymyxins mediated by Kir 4.2 and Kir5.1 plays a major role in polymyxin-induced nephrotoxicity.

In addition, apoptosis was also observed in the stroma and other tubular segments of the mouse kidney explant. This is very likely due to a limitation of mouse kidney explant culture, as all cell types (e.g. proximal and distal tubular cells) were exposed to polymyxins in culture media. However, in vivo after filtration by glomeruli, polymyxin concentration significantly increases in renal proximal tubule, where the interaction of polymyxin molecules with the Kir channels may lead to substantially higher intracellular uptake and toxicity in proximal tubular cells than in other renal cell populations (e.g. in distal tubule). Indeed, a recent in vitro study using human kidney organoids reported a similar broad cellular toxicity (e.g. in distal tubule and interstitial cell populations) of cisplatin, a chemotherapeutic agent which selectively targets proximal tubules in vivo [47]. Further studies are warranted to examine polymyxin impairment and the localisation of Kir 4.2 and Kir5.1 in other renal cell types with immunostaining.

In summary, we discovered several key genes mediating polymyxin-induced toxicity in human renal tubular HK-2 cells using whole-genome CRISPR screen in combination with transcriptomics validation. Our study is the first to reveal that the inwardly rectifying potassium channels Kir 4.2 (*KCNJ15*) and Kir5.1 (*KCNJ16*) mediate polymyxin-induced nephrotoxicity through membrane depolarization and cellular uptake. Our results will expedite the discovery of safer new-generation polymyxins that do not interact with these channels via a chemical biology approach. Importantly, our findings provide a novel approach to attenuate polymyxin-induced nephrotoxicity by targeting the inwardly rectifying potassium channels Kir 4.2 and Kir5.1, which may rescue their clinical utility against Gram-negative ‘superbugs’.

## Materials and methods

### Animal study approval

All animal studies were approved by Monash University Animal Ethics Committee MARP-2 under the approval number 22271.

### Chemicals and cell culture

Polymyxin B (Beta Pharma, Zhejiang, China) solution was prepared in Milli-Q™ water (Millipore, Melbourne, Australia) and filtered through 0.22-μm syringe filters (Sartorius, Melbourne, Australia). Cell viability was detected with XTT staining using 200 μg/mL XTT (Santa Cruz Biotechnology, Dallas, TX, USA) combined with 25 μM phenazine methosulfate (Sigma-Aldrich, Saint Louis, MO, USA) in culture medium for 2 h at 37 °C. Absorbance was measured at 475 and 660 nm using an Infinite M200 plate reader (Tecan group Ltd., Zürich, Switzerland). Mouse monoclonal anti-polymyxin B IgM antibody (Invitrogen, cat number: MA1-40133) and goat anti-mouse IgM conjugated with Alexa Fluor-594 were purchased from Thermo Fisher Scientific Australia Pty. Ltd (Melbourne, Australia). Rabbit multiclonal anti-Kir4.2 (KCNJ15) and anti-Kir5.1 (KCNJ16) antibodies were purchased from Alomone Labs (Jerusalem, Israel). The primary anti-actin antibody and goat anti-rabbit HRP-conjugated secondary antibody were produced from Sigma-Aldrich. All the other reagents were purchased from Sigma-Aldrich (Australia) and were of the highest commercial grade available unless otherwise stated.

Human kidney 2 (HK-2) cell line was purchased from American Type Culture Collection (Manassas, VA, USA) and cultured in keratinocyte serum free media supplemented with human recombinant epidermal growth factor 1–53 and bovine pituitary extract (KSFM, Invitrogen, Foster, CA, USA). Cells were incubated at 37 °C with 5% CO_2_.

### Pooled sgRNA libraries

The human Brunello pooled sgRNA library was used in this study (Addgene cat number: 73179) [17]. In total, there were 76,441 single guide RNAs (sgRNAs) targeting 19,114 genes [17] with approximately four sgRNAs targeting each gene plus 1000 non-targeting controls. The lentiviral vector with a puromycin resistance marker was employed to construct the sgRNA library [48].

### Construction of Cas9-expressing HK-2 cell line

To construct the Cas9-expressing cell line, HK-2 cells were infected with Cas9-containing lentivirus in the presence of 0.5 µg/mL polybrene. On the following day, the medium was replaced with fresh KSFM containing 4 µg/mL blasticidin. Culturing with blasticidin was continued for 6 days before Cas9 activity was validated with a lentiviral vector containing GFP and a sgRNA targeting GFP as reported [18].

### Genome-scale CRISPR-Cas9 knockout screen and data processing

CRISPR screening was carried out with the HK-2 cell line considering the following reasons. Firstly, HK-2 cells express the majority of the genes involved in kidney function [49]. Secondly, the HK-2 cell line is proliferative and amenable to genetic manipulation, which are essential to CRISPR screening and subsequent validations [17–19]. In our CRISPR screen, lentiviral particles containing the pooled sgRNA library were transduced to Cas9-expressing HK-2 cells with a multiplicity of infection (MOI) of 0.3 and a ratio of ~ 500 cells per sgRNA. Following transduction, infected cells were selected with 2 µg/mL puromycin for 14 days. The toxicity screen was performed in duplicates and each pooled mutant cell library replicate was divided into pre-drug, control and polymyxin B (treatment) groups. Polymyxin B (25 µM) was added to the treatment group to kill ~ 80% of cells, with the medium replenished once at day 7. After 14 days, cells were harvested from the control and treatment groups for genomic DNA extraction using NucleoSpin Blood XL (Clontech, Japan). The specific regions containing sgRNA were amplified for DNA sequencing using Illumina NextSeq500 as described [18]. SgRNA abundance was quantified using the PoolQ algorithm, and the MAGeCK algorithm [19] was used to identify sgRNAs enriched in these samples. The fold change of relative sgRNA abundance was determined by comparing the average log-transformed sgRNA abundance with and without polymyxin B treatment. Genes with a combination of FDR < 0.05 and FC ≥ 2 were considered as positive. Pathway enrichment was carried out with Reactome database [50].

### Gene knockout by CRISPR-Cas9 editing

Two sgRNAs for each gene were chosen and cloned into pXPR_BRD003 vector separately [18]. Top ranked genes *KCNJ15*, *KCNJ16*, *KEAP1*, *MAU2*, *NF2* and *TSC2* were selected for validation. Lentiviral vectors were produced in HEK293T cells after transfection with 1250 ng pXPR_BRD003 containing sgRNA, 250 ng VSVG, 1250 ng psPAX2 with Lipofectamine^®^ 2000 (Invitrogen). Viruses were collected at 48 h and freeze stocked at – 80 ℃ before being transduced into Cas9-expressing HK-2 cells. The transduced cells were then selected with 2 µg/mL puromycin. Viability of the gene knockout cells was measured with cell proliferation kit XTT after 24-h treatment with 25 µM polymyxin B.

### RNA-seq and data analysis

Total RNA of HK-2 samples was extracted using TRIzol Reagent (Invitrogen) and checked on NanoDrop (Thermo Fisher Scientific) and Agilent 2100 Bioanalyzer (Agilent Technologies, Santa Clara, CA, USA) before sequencing (150 bp paired-end) by Genewiz (Shanghai, China). Raw reads were aligned to human genome GRCh38.94 using subjunc [51] and summarized using featureCounts [52]. Genes with < 10 counts of aligned reads across all samples were excluded in analysis to reduce the noise [53]. Differentially expressed genes were identified using limma package [54] with a combination of fold change ≥ 1.5 and false discovery rate (FDR, by Benjamini–Hochberg algorithm) adjusted *p* value < 0.05. Gene set enrichment analysis was conducted using clusterProfiler [55, 56]. Functional relativeness between the CRISPR screen and gene differential expression results were examined by calculating gene semantic similarity using GOSemSim with GO terms [56].

### Western blot

Western blot was conducted as previously reported [57]. Specifically, wild-type, *KCNJ15* KO and *KCNJ16* KO HK-2 cells were collected with centrifugation. Cells (~ 1 × 10^6^) from each group were lysed with RIPA buffer (Invitrogen). Proteins were separated on a pre-casted SDS gel (Bio-Rad Hercules, CA, USA), and then transferred to a HybondTM-C Extra nitrocellulose membrane (Amersham Biosciences, Little Chalfont, UK). The primary antibodies for Kir4.2 (KCNJ15) and Kir5.1 (KCNJ16) were applied to detect the expression of both channels, and the primary actin antibody was used as an internal control. The membranes were visualized with Amersham ECL TM Prime Western Blotting Detection Reagents under an Amersham Imager 680 (GE Healthcare Bio-Sciences Corp, Marlborough, MA, USA).

### Functional validation of Kir4.2 and Kir5.1

HK-2 cells were dis-attached with trypsin and plated at 10,000 per well in a 96-well plate. Cells were treated with polymyxin B at 5–100 µM for 24 h and cell viability was measured with XTT. For the inhibitor experiments, cells were incubated with polymyxin B (25 µM), in combination with BaCl_2_ (5–200 µM), or VU0134992 (0–50 µM) for 24 h. Polymyxin B was added 10 min after the addition of the other agent unless otherwise stated and cell viability was measured with XTT. Data are presented as percentage to the respective untreated controls.

To examine polymyxin B uptake, HK-2 cells (wild-type and KOs) were plated 50,000 per well in a 24-well plate on 13 mm diameter round coverslips. After overnight attachment, cells were treated with 25 µM polymyxin B for 6 h, with or without BaCl_2_. The cells were then immuno-stained with mouse primary polymyxin antibody (Thermo Fisher Scientific) and goat anti-mouse Alexa Fluor-594 secondary antibody (Thermo Fisher Scientific), and the cell nuclei were counterstained with 4ʹ,6-diamidino-2-phenylindole (DAPI, Sigma-Aldrich). Images were taken using a Leica SP8 confocal microscope (Leica, Germany). The mean fluorescence intensity was measured by Image J [58] and the values were used after deducting from the non-treatment control. Four independent experiments were carried out.

To detect changes in cell membrane potential, HK-2, *KCNJ15* KO, *KCNJ16* KO cells were allowed to attach for 24 h before treatment with 25 µM polymyxin B for 1 h in a 24-well plate. DiBAC (20 pg/mL, Sigma–Aldrich) was added to each well and images were collected by a Leica DMi8 fluorescence microscope. The number of DiBAC-positive cells was measured with flow cytometry (NovoCyte, USA).

### Patch clamp electrophysiological measurements

Electrophysiological activity was measured via patch-clamp in whole-cell voltage clamp or current clamp mode using an Axopatch 200A series amplifier controlled by pCLAMP (version 10, Molecular Devices, USA). A coverslip containing HK-2 cells was placed in a recording bath (Warner Instruments, Hamden, CT, USA). The cells were continuously superfused at 1.5 mL/min at room temperature (25 °C) with Hanks balanced salt solution (HBSS, pH 7.4) containing NaCl 137 mM, NaHCO_3_ 4 mM, NaH_2_PO_4_ 0.3 mM, KCl 5.4 mM, KH_2_PO_4_ 0.44 mM, MgCl_2_ 0.5 mM, MgSO_4_ 0.4 mM, glucose 5.6 mM, HEPES 10 mM, and CaCl_2_ 1.5 mM. Recording pipettes (Clarke Glass, USA) were pulled using a micro-pipette puller (Flaming-Brown, Sutter Instruments, USA) and fire-polished to form a smooth tip (Narishige, Japan). The pipette tips had a resistance of 2.5–5 MΩ. Electrodes were filled with a solution containing KCl 130 mM, MgATP 2 mM, MgCl_2_ 1.2 mM, HEPES 10 mM, and EGTA 2 mM (pH 7.4). Cells were held at – 60 mV at the beginning and currents were recorded with voltage stepped from –120 to + 20 mV in 10 mV steps for 500 ms or when voltage ramps were applied (0.9 V/s).

Electrophysiological data were digitized at 5–20 kHz and analyzed using Clampfit 10 (Axon Instruments, USA). To determine the effect of polymyxin B, the current in the control solution was subtracted from the data recorded in the presence of polymyxin B at 50–250 µM. All data recorded are presented in the figures.

### All-atom molecular dynamics simulations of interactions between polymyxins and Kir4.2

A homology model of Kir4.2 channel was built in the SWISS-MODEL server based on the crystal structure of Kir2.2 channel (PDBID: 3SPI) [59]. CHARMM-GUI membrane builder module was employed to assemble the complex containing the Kir4.2 and DPPC bilayer [60]. SPC water model and 0.1 M potassium chloride were used to hydrate and neutralize the system. The simulation system contains approximately 270,000 atoms in total. In the simulations, 4 polymyxin B_1_ molecules were added in the top water layer by randomly replacing the water molecules. After energy minimization and six-step equilibration, 200-ns all-atom molecular dynamics simulations were conducted for each system to examine the conformational dynamics of Kir4.2 in the presence and absence of polymyxins. All molecular dynamics simulations were performed using GROMACS 2018 with the Monash University M3 MASSIVE supercomputer [61]. Other simulation parameters were set to match unbiased all-atom molecular dynamics simulations [62].

### Antibacterial activity of polymyxin B in the presence of Kir inhibitors

Activity of polymyxin B against *P. aeruginosa* PAO1, *A. baumannii* AB5075 and *K. pneumoniae* MKP103 was determined in the absence and presence of 50 μM BaCl_2_, or 5 μM VU0139942 using broth microdilution method [63]. Briefly, experiments were performed in 96-well polypropylene microtiter plates with concentrations of polymyxin B (0.125–64 mg/L) in cation-adjusted Mueller–Hinton broth (Oxoid, UK). The lowest concentrations that inhibited visible bacterial growth after a 20-h incubation at 37 °C were compared.

### Kidney explant culture and treatments

Kidneys were harvested on embryonic day E13.5 from C57BL/6 J mice and cultured on a transwell insert in a 6-well plate in DMEM with 10% FCS for 24 h as previously reported [29, 30]. The medium was then replaced with fresh medium containing (i) polymyxin B (25, 50, or 100 µM), (ii) 5 µM VU0134992 alone, (iii) 50 µM BaCl_2_ alone, (iv) 5 µM VU0134992 plus 50 µM polymyxin B, or (v) 50 µM BaCl_2_ plus 50 µM polymyxin B. Treated kidneys were cultured for an extra 20–24 h, taking them to a developmental stage approximately equivalent to E15.5. A subset of kidney cultures were stained with propidium iodide (PI) and Hoechst 33342 for 10 min, then washed three times and fixed in 4% polyformaldehyde (PFA) for imaging. For the TUNEL assay, samples were fixed in 4% PFA for 10 min and stained with Click-iT™ TUNEL Alexa Fluor™ 647 Imaging Assay (Molecular Probes, Thermo Fisher Scientific). Proximal tubules were detected using biotinylated Lotus Tetragonolobus Lectin (LTL, Vector Laboratories, Inc. Burlingame, CA, USA) and were revealed with fluorescence-labelled streptavidin (Invitrogen). Nuclei were counterstained with DAPI. Kidneys labelled with the TUNEL assay, DAPI and LTL, were mounted in 50% (v/v) glycerol on a glass bottom dish and images were taken using AS 980 Zeiss at 40 × oil immersion with a *z*-stack between 15 and 35 sections at a 2-μm step-size (30 to 70 µm thick). Quantification was conducted in the following steps: (i) calculation of the total number of apoptotic (TUNNEL-positive) cells and the number of total (DAPI-positive) cells using the 3D counter and 3D iterative threshold plugins in Image J, respectively [64, 65]; (ii) For calculation of the same parameters as above in the tubules, the proximal tubule outline (LTL-positive) was drawn on each image to create a binary mask, and the 3D counter plugin and iterative threshold plugin were used for counting TUNEL-positive and DAPI-positive cells, respectively. The representative images in Fig. 7A are flattened stacks (maximum intensity projections) of four slices for each condition (8-μm in total). Due to the intensity disparity of apoptotic cells in the tissue, the TUNEL channel was binarized to facilitate visualization of the apoptotic cells.

### Expression levels of *Kcnj15* and *Kcnj16* in kidneys of fetal mice

The expression data of E18.5 were generated and processed as previously described and are available at the Gene Expression Omnibus database (GSE108291) [66]. Embryonic E15.5 kidneys were dissected and dissociated in 500 μL ACCUTASE™ (Stemcell Technologies, Vancouver, Canada) at 37 °C for 6–8 min. Samples were gently agitated by pipetting every 2 min, washed with cold phosphate-buffered saline (PBS) containing 0.05% bovine serum albumin (BSA), and pelleted by centrifugation (400 *g*, 5 min). Cell concentration was determined using a hemocytometer and adjusted prior to the generation of single-cell libraries using 10 × Chromium v3 kits (10 × Genomics). Samples were sequenced at Murdoch Children’s Research Institute, Australia. Sequencing data were processed using Cell Ranger (v1.3.1, 10 × Genomics) and aligned to mm10 with STAR (v2.5.1b) [67]. Subsequent analysis was performed in R using Seurat (v3.1.4) [68]. Quality control for the E15.5 dataset involved the removal of cells with < 1500 genes, or > 8% mitochondrial gene content. Doublets were identified and filtered out using Scrublet [69] or the HTODemux function in Seurat [70]. Following all quality control steps, the E15.5 dataset consisted of 18,549 genes and 3294 cells. Cell cycle effects were regressed out and gene expression data were normalized using SCTransform, followed by clustering at resolution 0.8. Cluster identity was determined as per to our previous analysis [66] then manually curated to generate 13 broad clusters comparable between the two developmental time-points. The E15.5 dataset is available upon request. Seurat’s DotPlot and FeaturePlot functions were employed for plotting and visualization. The average expression scale was used to compare expression levels between different clusters. The values were scaled such that 0 represents the mean expression of the gene across the whole dataset and a value of 2 shows that the gene expression in that cluster is two standard deviations above the mean. For percent expression, it shows the percentage of cells in that cluster which expressed the gene of interest.

### Statistical analysis

Data analysis and graphing were performed with GraphPad Prism 9.0 (GraphPad Software Inc., San Diego, CA, USA), unless otherwise stated. Data are presented as box and whisker plots and were normally distributed with homogeneous variance as determined by Shapiro–Wilk normality test. One-way or two-way analysis of variance (ANOVA) were employed for multi-group comparisons, followed by Tukey's multiple comparison test for *p* values. *p < 0.05; **p < 0.01; ***p < 0.001; ****p < 0.0001.

## Supplementary Information

Below is the link to the electronic supplementary material.

Supplementary file1 (DOCX 6503 KB)

Data file S1. sgRNA enrichment in CRISPR screening in HK-2 cells with 25 µM polymyxin B treatment for 14 days. This file includes read counts of sgRNAs (Worksheet 1) and gene rank with fold change and adjusted p-value (Worksheet 2)

Data file S2. Differentially expressed genes in HK-2 cells with or without 100 µM polymyxin B treatment for 24 h (FDR < 0.5 and fold change > 1.5)

## Data Availability

The data that support the findings of this study are available from the corresponding author on reasonable request.

## References

[1] Lehtinen S, Blanquart F, Lipsitch M, Fraser C (2019). On the evolutionary ecology of multidrug resistance in bacteria. PLoS Pathog.

[2] Velkov T, Thompson PE, Azad MAK, Roberts KD, Bergen PJ (2019). History, chemistry and antibacterial spectrum. Adv Exp Med Biol.

[3] Li J, Nation RL, Turnidge JD, Milne RW, Coulthard K (2006). Colistin: the re-emerging antibiotic for multidrug-resistant Gram-negative bacterial infections. Lancet Infect Dis.

[4] Poirel L, Jayol A, Nordmann P (2017). Polymyxins: antibacterial activity, susceptibility testing, and resistance mechanisms encoded by plasmids or chromosomes. Clin Microbiol Rev.

[5] Tsuji BT, Pogue JM, Zavascki AP, Paul M, Daikos GL (2019). International consensus guidelines for the optimal use of the polymyxins: endorsed by the American College of Clinical Pharmacy (ACCP), European Society of Clinical Microbiology and Infectious Diseases (ESCMID), Infectious Diseases Society of America (IDSA), International Society for Anti-infective Pharmacology (ISAP), Society of Critical Care Medicine (SCCM), and Society of Infectious Diseases Pharmacists (SIDP). Pharmacotherapy.

[6] Nang SC, Azad MAK, Velkov T, Zhou QT, Li J (2021). Rescuing the last-line polymyxins: achievements and challenges. Pharmacol Rev.

[7] Zavascki AP, Nation RL (2017). Nephrotoxicity of polymyxins: is there any difference between colistimethate and polymyxin B?. Antimicrob Agents Chemother.

[8] Hartzell JD, Neff R, Ake J, Howard R, Olson S (2009). Nephrotoxicity associated with intravenous colistin (colistimethate sodium) treatment at a tertiary care medical center. Clin Infect Dis.

[9] Ghlissi Z, Hakim A, Mnif H, Ayadi FM, Zeghal K (2013). Evaluation of colistin nephrotoxicity administered at different doses in the rat model. Ren Fail.

[10] Abdelraouf K, Braggs KH, Yin T, Truong LD, Hu M (2012). Characterization of polymyxin B-induced nephrotoxicity: implications for dosing regimen design. Antimicrob Agents Chemother.

[11] Dai C, Li J, Tang S, Li J, Xiao X (2014). Colistin-induced nephrotoxicity in mice involves the mitochondrial, death receptor, and endoplasmic reticulum pathways. Antimicrob Agents Chemother.

[12] Azad MA, Akter J, Rogers KL, Nation RL, Velkov T (2015). Major pathways of polymyxin-induced apoptosis in rat kidney proximal tubular cells. Antimicrob Agents Chemother.

[13] Yun B, Zhang T, Azad MAK, Wang J, Nowell CJ (2018). Polymyxin B causes DNA damage in HK-2 cells and mice. Arch Toxicol.

[14] Forrest A, Garonzik SM, Thamlikitkul V, Giamarellos-Bourboulis EJ, Paterson DL (2017). Pharmacokinetic/toxicodynamic analysis of colistin-associated acute kidney injury in critically ill patients. Antimicrob Agents Chemother.

[15] Sandri AM, Landersdorfer CB, Jacob J, Boniatti MM, Dalarosa MG (2013). Population pharmacokinetics of intravenous polymyxin B in critically ill patients: implications for selection of dosage regimens. Clin Infect Dis.

[16] Azad MA, Roberts KD, Yu HH, Liu B, Schofield AV (2015). Significant accumulation of polymyxin in single renal tubular cells: a medicinal chemistry and triple correlative microscopy approach. Anal Chem.

[17] Doench JG, Fusi N, Sullender M, Hegde M, Vaimberg EW (2016). Optimized sgRNA design to maximize activity and minimize off-target effects of CRISPR-Cas9. Nat Biotechnol.

[18] Rosenbluh J, Xu H, Harrington W, Gill S, Wang X (2017). Complementary information derived from CRISPR Cas9 mediated gene deletion and suppression. Nat Commun.

[19] Li W, Xu H, Xiao T, Cong L, Love MI (2014). MAGeCK enables robust identification of essential genes from genome-scale CRISPR/Cas9 knockout screens. Genome Biol.

[20] Ahmed MU, Velkov T, Lin YW, Yun B, Nowell CJ (2017). Potential toxicity of polymyxins in human lung epithelial cells. Antimicrob Agents Chemother.

[21] Lambertz N, El Hindy N, Kreitschmann-Andermahr I, Stein KP, Dammann P (2015). Downregulation of programmed cell death 10 is associated with tumor cell proliferation, hyperangiogenesis and peritumoral edema in human glioblastoma. BMC Cancer.

[22] Chen L, Tanriover G, Yano H, Friedlander R, Louvi A (2009). Apoptotic functions of PDCD10/CCM3, the gene mutated in cerebral cavernous malformation 3. Stroke.

[23] Bignon Y, Pinelli L, Frachon N, Lahuna O, Figueres L (2020). Defective bicarbonate reabsorption in Kir4.2 potassium channel deficient mice impairs acid-base balance and ammonia excretion. Kidney Int.

[24] Paulais M, Bloch-Faure M, Picard N, Jacques T, Ramakrishnan SK (2011). Renal phenotype in mice lacking the Kir5.1 *(KCNJ16)* K^+^ channel subunit contrasts with that observed in SeSAME/EAST syndrome. Proc Natl Acad Sci U S A.

[25] Hibino H, Inanobe A, Furutani K, Murakami S, Findlay I (2010). Inwardly rectifying potassium channels: their structure, function, and physiological roles. Physiol Rev.

[26] Alagem N, Dvir M, Reuveny E (2001). Mechanism of Ba^2+^ block of a mouse inwardly rectifying K^+^ channel: differential contribution by two discrete residues. J Physiol.

[27] Kharade SV, Kurata H, Bender AM, Blobaum AL, Figueroa EE (2018). Discovery, characterization, and effects on renal fluid and electrolyte excretion of the Kir4.1 potassium channel pore blocker, VU0134992. Mol Pharmacol.

[28] Duan XP, Gu L, Xiao Y, Gao ZX, Wu P (2019). Norepinephrine-induced stimulation of Kir4.1/Kir5.1 is required for the activation of NaCl transporter in distal convoluted tubule. Hypertension.

[29] Combes AN, Lefevre JG, Wilson S, Hamilton NA, Little MH (2016). Cap mesenchyme cell swarming during kidney development is influenced by attraction, repulsion, and adhesion to the ureteric tip. Dev Biol.

[30] Lawlor KT, Zappia L, Lefevre J, Park JS, Hamilton NA et al (2019) Nephron progenitor commitment is a stochastic process influenced by cell migration. eLife 24;8:e41156 10.7554/eLife.41156 PMC6363379 30676318

[31] Nation RL, Garonzik SM, Thamlikitkul V, Giamarellos-Bourboulis EJ, Forrest A (2017). Dosing guidance for intravenous colistin in critically-ill patients. Clin Infect Dis.

[32] Nelson BC, Eiras DP, Gomez-Simmonds A, Loo AS, Satlin MJ (2015). Clinical outcomes associated with polymyxin B dose in patients with bloodstream infections due to carbapenem-resistant Gram-negative rods. Antimicrob Agents Chemother.

[33] Pearson WL, Dourado M, Schreiber M, Salkoff L, Nichols CG (1999). Expression of a functional Kir4 family inward rectifier K+ channel from a gene cloned from mouse liver. J Physiol.

[34] Moran-Zendejas R, Delgado-Ramirez M, Xu J, Valdes-Abadia B, Arechiga-Figueroa IA (2020). In vitro and in silico characterization of the inhibition of Kir4.1 channels by aminoglycoside antibiotics. Br J Pharmacol.

[35] Tao X, Avalos JL, Chen J, MacKinnon R (2009). Crystal structure of the eukaryotic strong inward-rectifier K+ channel Kir2.2 at 3.1 A resolution. Science.

[36] Perazella MA (2018). Pharmacology behind common drug nephrotoxicities. Clin J Am Soc Nephrol.

[37] Suzuki T, Yamaguchi H, Ogura J, Kobayashi M, Yamada T (2013). Megalin contributes to kidney accumulation and nephrotoxicity of colistin. Antimicrob Agents Chemother.

[38] Lu X, Chan T, Xu C, Zhu L, Zhou QT (2016). Human oligopeptide transporter 2 (PEPT2) mediates cellular uptake of polymyxins. J Antimicrob Chemother.

[39] Visentin M, Gai ZB, Torozi A, Hiller C, Kullak-Ublick GA (2017). Colistin is substrate of the carnitine/organic cation transporter 2 (OCTN2, SLC22A5). Drug Metab Dispos.

[40] Ahmed MU, Velkov T, Zhou QT, Fulcher AJ, Callaghan J (2019). Intracellular localization of polymyxins in human alveolar epithelial cells. J Antimicrob Chemother.

[41] Saxton RA, Sabatini DM (2017). mTOR signaling in growth, metabolism, and disease. Cell.

[42] Park J, Shrestha R, Qiu C, Kondo A, Huang S (2018). Single-cell transcriptomics of the mouse kidney reveals potential cellular targets of kidney disease. Science.

[43] Ding F, Tian X, Mo J, Wang B, Zheng J (2021). Determination of the dynamic cellular transcriptional profiles during kidney development from birth to maturity in rats by single-cell RNA sequencing. Cell Death Discov.

[44] Muto Y, Wilson PC, Ledru N, Wu H, Dimke H (2021). Single cell transcriptional and chromatin accessibility profiling redefine cellular heterogeneity in the adult human kidney. Nat Commun.

[45] Manchandani P, Zhou J, Ledesma KR, Truong LD, Chow DS (2016). Characterization of polymyxin B biodistribution and disposition in an animal model. Antimicrob Agents Chemother.

[46] Yun B, Azad MA, Wang J, Nation RL, Thompson PE (2015). Imaging the distribution of polymyxins in the kidney. J Antimicrob Chemother.

[47] Digby JLM, Vanichapol T, Przepiorski A, Davidson AJ, Sander V (2020). Evaluation of cisplatin-induced injury in human kidney organoids. Am J Physiol Renal Physiol.

[48] Joung J, Konermann S, Gootenberg JS, Abudayyeh OO, Platt RJ (2017). Genome-scale CRISPR-Cas9 knockout and transcriptional activation screening. Nat Protoc.

[49] Wanic K, Krolewski B, Ju W, Placha G, Niewczas MA (2013). Transcriptome analysis of proximal tubular cells (HK-2) exposed to urines of type 1 diabetes patients at risk of early progressive renal function decline. PLoS ONE.

[50] Jassal B, Matthews L, Viteri G, Gong C, Lorente P (2020). The reactome pathway knowledgebase. Nucleic Acids Res.

[51] Liao Y, Smyth GK, Shi W (2019). The R package Rsubread is easier, faster, cheaper and better for alignment and quantification of RNA sequencing reads. Nucleic Acids Res.

[52] Liao Y, Smyth GK, Shi W (2014). featureCounts: an efficient general purpose program for assigning sequence reads to genomic features. Bioinformatics.

[53] Koch CM, Chiu SF, Akbarpour M, Bharat A, Ridge KM (2018). A Beginner's guide to analysis of RNA sequencing data. Am J Respir Cell Mol Biol.

[54] Ritchie ME, Phipson B, Wu D, Hu Y, Law CW (2015). limma powers differential expression analyses for RNA-sequencing and microarray studies. Nucleic Acids Res.

[55] Kuleshov MV, Jones MR, Rouillard AD, Fernandez NF, Duan Q (2016). Enrichr: a comprehensive gene set enrichment analysis web server 2016 update. Nucleic Acids Res.

[56] Jin X, Yu L, Wu Y, Zhang S, Shi Z (2012). S-Glutathionylation underscores the modulation of the heteromeric Kir4.1-Kir5.1 channel in oxidative stress. J Physiol.

[57] Duan J, Lu G, Xie Z, Lou M, Luo J (2014). Genome-wide identification of CRISPR/Cas9 off-targets in human genome. Cell Res.

[58] Schindelin J, Arganda-Carreras I, Frise E, Kaynig V, Longair M (2012). Fiji: an open-source platform for biological-image analysis. Nat Methods.

[59] Arechiga-Figueroa IA, Marmolejo-Murillo LG, Cui M, Delgado-Ramirez M, van der Heyden MAG (2017). High-potency block of Kir4.1 channels by pentamidine: molecular basis. Eur J Pharmacol.

[60] Wu EL, Cheng X, Jo S, Rui H, Song KC (2014). CHARMM-GUI Membrane Builder toward realistic biological membrane simulations. J Comput Chem.

[61] Van Der Spoel D, Lindahl E, Hess B, Groenhof G, Mark AE (2005). GROMACS: fast, flexible, and free. J Comput Chem.

[62] Jiang X, Li W, Chen G, Wang L (2017). Dynamic perturbation of the active site determines reversible thermal inactivation in glycoside hydrolase family 12. J Chem Inf Model.

[63] Luber P, Bartelt E, Genschow E, Wagner J, Hahn H (2003). Comparison of broth microdilution, E Test, and agar dilution methods for antibiotic susceptibility testing of Campylobacter jejuni and Campylobacter coli. J Clin Microbiol.

[64] Bolte S, Cordelieres FP (2006). A guided tour into subcellular colocalization analysis in light microscopy. J Microsc.

[65] Ollion J, Cochennec J, Loll F, Escude C, Boudier T (2013). TANGO: a generic tool for high-throughput 3D image analysis for studying nuclear organization. Bioinformatics.

[66] Combes AN, Phipson B, Lawlor KT, Dorison A, Patrick R (2019). Single cell analysis of the developing mouse kidney provides deeper insight into marker gene expression and ligand-receptor crosstalk. Development.

[67] Dobin A, Davis CA, Schlesinger F, Drenkow J, Zaleski C (2013). STAR: ultrafast universal RNA-seq aligner. Bioinformatics.

[68] Stuart T, Butler A, Hoffman P, Hafemeister C, Papalexi E (2019). Comprehensive integration of single-cell data. Cell.

[69] Wolock SL, Lopez R, Klein AM (2019). Scrublet: computational identification of cell doublets in single-cell transcriptomic data. Cell Syst.

[70] Scialdone A, Natarajan KN, Saraiva LR, Proserpio V, Teichmann SA (2015). Computational assignment of cell-cycle stage from single-cell transcriptome data. Methods.

