# Supplementary material for: Inwardly rectifying potassium channels mediate polymyxin-induced nephrotoxicity
Source: Cell Mol Life Sci. 2022 May 15;79(6):296. doi: 10.1007/s00018-022-04316-z (PMC9108107; doi:10.1007/s00018-022-04316-z)
Supplement: Supplementary file 1 — Supplementary file1 (DOCX 6503 KB) [file 18_2022_4316_MOESM1_ESM.docx]

**Supplementary Information for**

**Inwardly rectifying potassium channels mediate polymyxin-induced nephrotoxicity**

Jing Lu,^1^ Mohammad A. K. Azad,^1,†^ Julie L. M. Moreau,^2,†^ Yan Zhu,^1^ Xukai Jiang,^1,3^ Mary Tonta,^4^ Rachel Lam,^2^ Hasini Wickremasinghe,^1^ Jinxin Zhao,^1^ Jiping Wang,^1^ Harold A. Coleman,^4^ Luke E. Formosa,^5^ Tony Velkov,^6^ Helena C. Parkington,^4^ Alexander N. Combes,^2^ Joseph Rosenbluh,^5,7^ Jian Li^1,*^

*Correspondence to: jian.li@monash.edu.

†Equal contribution.

**This file includes:**

Supplementary figures

**Supplementary Figures 1 to 3**

**Supplementary table**

**Supplementary Table 1**

**Titles for Data files**

**Data file S1**

**Data file S2**

Supplementary figures

Supplementary Figure 1


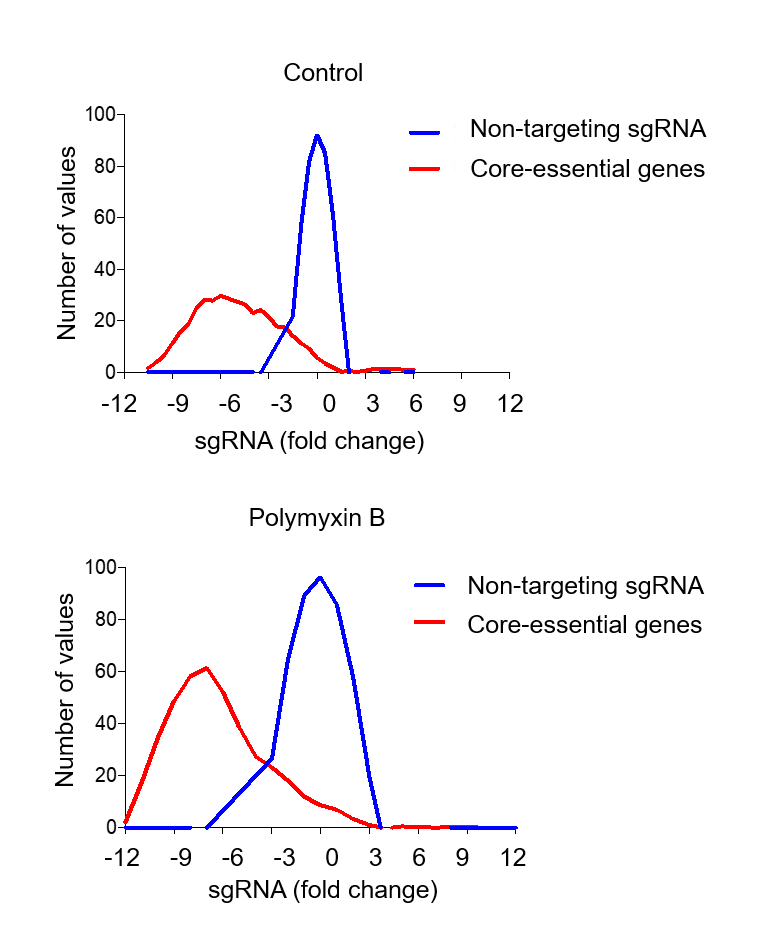


**Figure S1. Changes of essential gene sgRNAs during the CRISPR screening in the control and polymyxin B treatment groups.**

Supplementary Figure 2

Figure S2. Gene semantic similarity for each pair of a CRISPR-identified gene and differentially expressed gene. (A-C) CRISPR-identified gene upon knockout conferred resistance to polymyxin B, and a differentially expressed gene in response to polymyxin B treatment in the semantic context of GO cellular component (A), molecular function (B), and biological process (C), respectively.

**Supplementary Figure 3**


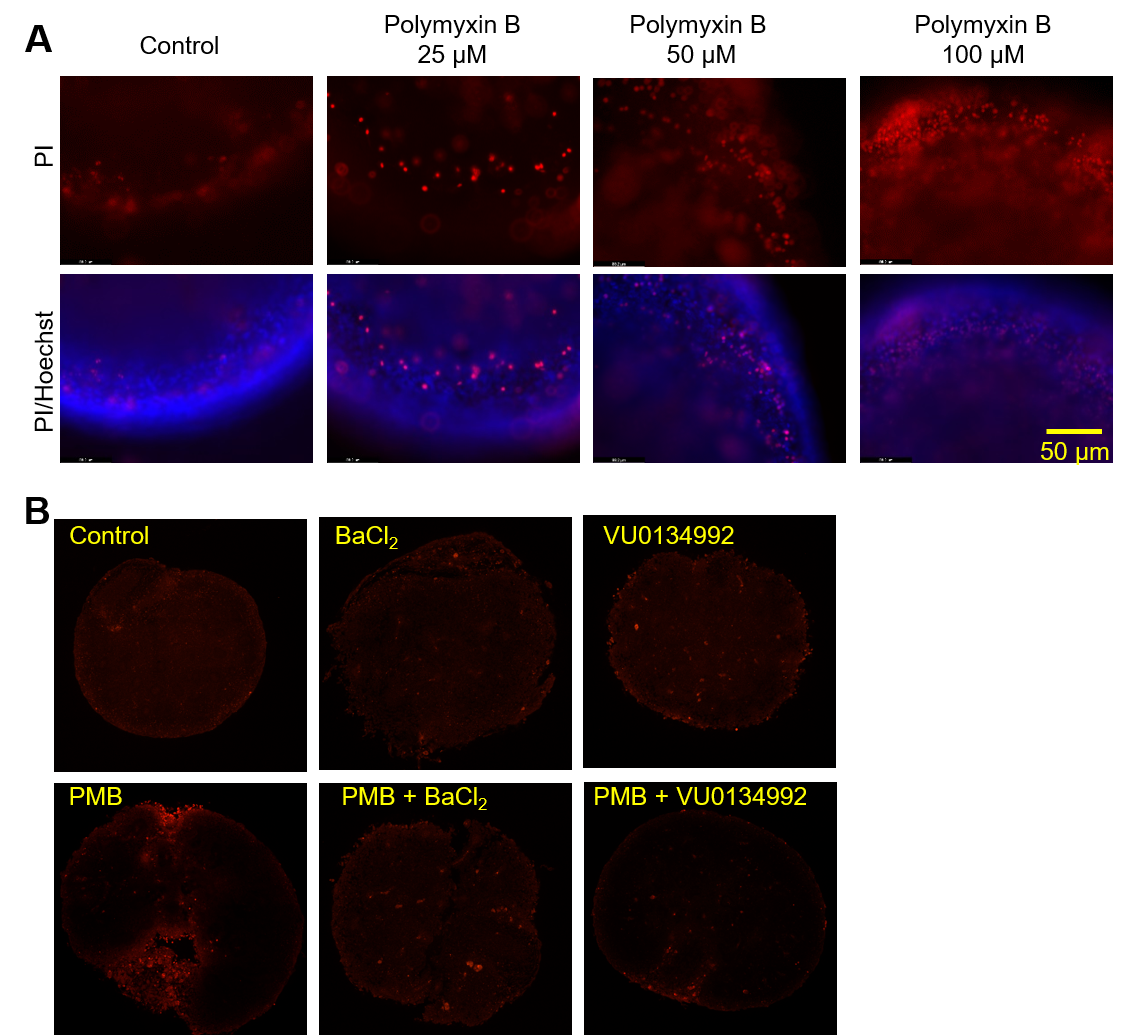


**Figure S3. Cell death in kidney explant culture after polymyxin B treatment with and without BaCl_2_ or VU0134992. (A)** Propidium iodide (PI, red) staining showing cell death in explant kidney culture after treatment with polymyxin B at 25, 50, and 100 µM (*n* = 3). Cell nuclei were counterstained with Hoechst 33342 (blue). **(B)** PI staining showing cell death in kidneys treated with 50 µM polymyxin B (PMB) alone or in combination with 50 µM BaCl_2_ and 5 µM VU0134922 (VU) (*n* = 3).

**Supplementary Table**

**Supplementary Table 1.** Minimum inhibitory concentrations (mg/L) of polymyxin B against *P. aeruginosa* PAO1, *A. baumannii* AB5075 and *K. pneumoniae* MKP103 with or without 50 μM BaCl_2_, or 5 μM VU0139942.

|  | **Polymyxin B** | **Polymyxin B**  **+ 50 μM BaCl_2_** | **Polymyxin B**  **+ 5 μM VU013994** |
| --- | --- | --- | --- |
| ***P. aeruginosa* PAO1** | 1 | 1 | 1 |
| ***A. baumannii* AB5075** | 0.5 | 0.5 | 0.5 |
| ***K. pneumoniae* MKP103** | 8 | 8 | 8 |
